# Factors affecting the provision of analgesia during childbirth, Japan

**DOI:** 10.2471/BLT.19.230128

**Published:** 2019-07-17

**Authors:** Yuto Maeda, Kenzo Takahashi, Kana Yamamoto, Tetsuya Tanimoto, Masahiro Kami, Andy Crump

**Affiliations:** a National Center of Child Health and Development, 2-10-1 Okura, Setagaya-ku, Tokyo, 157-8535, Japan.; b Teikyo University Graduate School of Public Health, Tokyo, Japan.; c Medical Governance Research Institute, Tokyo, Japan.; d Navitas Clinic, Tokyo, Japan.; e Kitasato University, Tokyo, Japan.

## Abstract

Japan’s universal health-care system means that it is a very safe country in which to give birth. Perinatal outcomes in Japan are excellent, with low infant mortality and neonatal mortality. However, childbirth remains a challenge for many Japanese women, who are faced with a scarcity of places to give birth, limited availability of analgesia and social norms that favour natural birth. The number of birth facilities in Japan continues to decrease as fewer children are born. The numbers of qualified medical staff remain inadequate, with a continuing lack of female physicians, perpetuated by a pervasive negative gender bias. Recruitment efforts are underway, but few doctors want to specialize in obstetrics or gynaecology. Furthermore, around half of female obstetricians and gynaecologists in Japan’s male-dominated medical system stop practising when they have their own children. The difficulty of obtaining analgesia during labour is another problem. Although low uptake of labour pain relief in Japan is said to be due to cultural influences, the root of the problem is a lack of qualified anaesthesiologists and the inflexibility of a system that will not allow other staff to be trained to administer labour analgesia. Problems with labour anaesthesia have been linked to 14 maternal deaths since 2010. Japanese policy-makers need to act to renovate the nation’s obstetric facilities, reorganize regional perinatal care systems, train more obstetricians and anaesthesiologists, promote task-shifting and better integrate biomedical and traditional, non-medical care for pregnant women.

## Résumé

Le système de soins de santé universel du Japon en fait un pays très sûr pour les accouchements. Les résultats en matière de santé périnatale au Japon sont excellents, avec une mortalité infantile et une mortalité néonatale faibles. Néanmoins, l’accouchement reste problématique pour de nombreuses Japonaises, qui doivent faire face à un manque d’établissements adaptés pour les accouchements, à une disponibilité limitée de l’analgésie et à des normes sociales qui favorisent les accouchements naturels. Le nombre de maternités au Japon ne cesse de baisser, à mesure de la diminution du nombre de naissances. Les effectifs en personnel médical qualifié restent inappropriés, avec une pénurie persistante de femmes médecins, perpétuée par des préjugés négatifs généralisés liés au genre. Des efforts de recrutement sont en cours, mais peu de médecins souhaitent se spécialiser en obstétrique ou en gynécologie. Par ailleurs, dans le système médical japonais dominé par les hommes, près de la moitié des femmes obstétriciennes et gynécologues cessent de pratiquer dès qu’elles ont elles-mêmes des enfants. La difficulté à bénéficier d’une analgésie obstétricale pendant l’accouchement constitue un autre problème. Même si, au Japon, le faible recours au soulagement de la douleur pendant le travail est généralement imputé à des influences culturelles, le fond du problème est une pénurie d’anesthésistes qualifiés et le manque de souplesse d’un système qui ne permet pas de former d’autres professionnels de santé pour pouvoir pratiquer l’analgésie obstétricale. Les problèmes liés à la réalisation d’une analgésie obstétricale ont donné lieu à 14 décès maternels depuis 2010. Au Japon, les décideurs politiques doivent agir pour rénover les installations obstétricales du pays, réorganiser les systèmes régionaux de santé périnatale, former davantage d’obstétriciens et d’anesthésistes, promouvoir le transfert des tâches et mieux intégrer les soins biomédicaux et les soins traditionnels, non médicaux, pour les femmes enceintes.

## Resumen

El sistema universal de atención sanitaria de Japón hace que sea un país muy seguro para dar a luz. Los resultados perinatales en Japón son excelentes, con baja mortalidad infantil y mortalidad neonatal. Sin embargo, el parto sigue siendo un problema para muchas mujeres japonesas, que se enfrentan a la escasez de lugares para dar a luz, la limitada disponibilidad de analgesia y las normas sociales que favorecen el parto natural. El número de centros de maternidad en Japón sigue disminuyendo a medida que nacen menos niños. El número de personal médico cualificado sigue siendo insuficiente, con una continua falta de mujeres médicas, perpetuada por un prejuicio de género negativo generalizado. Los esfuerzos de reclutamiento están en marcha, pero pocos médicos quieren especializarse en obstetricia o ginecología. Además, alrededor de la mitad de las obstetras y ginecólogas del sistema médico japonés dominado por los hombres dejan de ejercer cuando tienen sus propios hijos. La dificultad para obtener analgesia durante el trabajo de parto es otro problema. Aunque se dice que la baja aceptación del alivio del dolor del trabajo de parto en Japón se debe a influencias culturales, la raíz del problema es la falta de anestesiólogos calificados y la inflexibilidad de un sistema que no permitirá que otro personal esté capacitado para administrar la analgesia del trabajo de parto. Los problemas con la anestesia del trabajo de parto se han relacionado con 14 muertes maternas desde 2010. Los responsables de formular políticas en Japón deben actuar para renovar las instalaciones obstétricas del país, reorganizar los sistemas regionales de atención perinatal, capacitar a más obstetras y anestesiólogos, promover el cambio de tareas e integrar mejor la atención biomédica y la atención no médica tradicional para las mujeres embarazadas.

## ملخص

إن وجود نظام عالمي للرعاية الصحية في اليابان يعني أنها دولة آمنة للغاية لعمليات الولادة. حيث تكون النتائج المتعلقة بالفترة المحيطة بالولادة في اليابان ممتازة، مع انخفاض معدل الوفيات لكل من الرضع والأطفال حديثي الولادة. ورغم ذلك، إلا أن الولادة لا تزال تمثل تحدياً للعديد من النساء اليابانيات، اللاتي يواجهن ندرة في أماكن الولادة، وتوافر محدود لمستلزمات التخدير، وكذلك التقاليد الاجتماعية التي تفضل الولادة الطبيعية. يستمر عدد مرافق الولادة في اليابان في الانخفاض بالتوازي مع انخفاض عدد الأطفال الذين يولدون. كذلك لا يزال عدد الطواقم الطبية المؤهلة غير كاف، مع نقص متواصل في الطبيبات الإناث، والذي أدى إليه التحيز السلبي بناء على الجنس. لا زالت هناك جهود جارية، إلا أن القليل من الأطباء يرغبون في التخصص في طب التوليد أو أمراض النساء. وفضلاً عن ذلك، فإن قرابة النصف من الطبيبات الإناث في طب التوليد وأمراض النساء في النظام الطبي الياباني الذي يخضع لسيطرة الذكور، يتوقفن عن ممارسة المهنة عندما يلدن أطفالهن. تمثل صعوبة الحصول على التخدير أثناء الولادة، مشكلة أخرى. على الرغم من القول بأن انخفاض معدل الحصول على مسكنات لآلام الولادة في اليابان هو بسبب المعتقدات الثقافية، فإن أصل المشكلة يعود لعدم وجود أطباء التخدير المؤهلين وعدم مرونة النظام الذي لا يسمح بتدريب العاملين الآخرين على التخدير قبل الولادة. تم الربط بين مشكلات التخدير أثناء الولادة وبين 14 حالة وفاة للأمهات منذ عام 2010. يحتاج واضعو السياسات اليابانيون إلى السعي لتجديد مرافق الولادة في الدولة، إعادة تنظيم نظم الرعاية الإقليمية في الفترة المحيطة بالولادة، وتدريب المزيد من أطباء التوليد وأطباء التخدير، والتشجيع على تغيير المهام، وتحسين التكامل بين الطب الحيوي والرعاية التقليدية غير الطبية للنساء الحوامل.

## 摘要

日本的全民医疗体系保证了在该国分娩的安全。日本的围产儿预后很好，婴儿死亡率和新生儿死亡率都很低。然而，对多数日本妇女来说，分娩仍是一种挑战，她们面临着医院床位紧张、分娩镇痛措施有限以及该国更倾向于自然生产的社会准则。随着日本新生儿数量的下降，该国可提供分娩措施的医疗机构数量也在持续下降。可以胜任的医务人员数量不足，由于社会上普遍存在的负面性别偏见，一直以来，女医生的数量尤其匮乏。人才招募方面的努力仍在持续，但很少有医生想专攻妇产科。此外，在日本男性占主导地位的医疗体系中，约一半的女性妇产科医生在有了自己的孩子后停止执业。另一大难题是分娩过程中难以进行镇痛措施。尽管将日本社会对分娩镇痛的接受度低归结为文化影响，但其根源在于缺乏可以胜任的麻醉师，以及僵化的医疗体系（不允许其他工作人员接受分娩镇痛培训）。自 2010 年以来，分娩镇痛带来的问题已经与 14 例产妇死亡相关。日本的相关政策制订者需采取行动，修复全国范围内可提供分娩措施的医疗机构，重新组织区域性的围产儿医疗体系，培训更多的产科医生和麻醉师，促进任务交付原则，将生物医学和传统的非医疗保健相结合，为孕产妇提供更好的护理。

## Резюме

Всеобщая система здравоохранения в Японии построена таким образом, что роды в этой стране отличаются высоким уровнем безопасности. Уровень благоприятных перинатальных исходов в Японии крайне высок, а смертность новорожденных и младенцев очень низкая. Однако для многих японок роды остаются проблемой из-за того, что количество родильных домов ограниченно, возможности обезболивания невелики, а социальные нормы поддерживают естественное родоразрешение. Количество родильных домов в Японии продолжает сокращаться в связи с падением рождаемости. Сохраняется проблема нехватки квалифицированного медперсонала, постоянно недостает женщин-врачей. Эта ситуация сохраняется из-за всеобъемлющего гендерного неравенства в стране. Конечно, предпринимаются усилия по привлечению кадров, но очень немногие врачи проявляют желание специализироваться в области акушерства и гинекологии. Кроме того, примерно половина акушерок и женщин-гинекологов, работающих в системе здравоохранения Японии, где доминируют мужчины, уходит из профессии с появлением у них собственных детей. Еще одна проблема — это сложности с обезболиванием при родах. Хотя низкий уровень обезболивания при родах обычно объясняют культурными особенностями Японии, проблема коренится в недостатке квалифицированных анестезиологов и в ригидности системы, которая не разрешает другому медперсоналу пройти обучение и выполнять обезболивание во время родов. Проблемы с обезболиванием при родах связывают с гибелью 14 рожениц с 2010 года. Высшему руководству Японии следует предпринять меры по обновлению национальной сети родильных домов, реорганизовать региональные системы перинатальной помощи, обучить больше акушерского персонала и анестезиологов, поощрять многозадачность персонала и лучше объединять биомедицинские и традиционные, немедицинские способы ухода за беременными женщинами.

## Introduction

At 1.44 births per woman in 2016, Japan’s low birth rate is an issue for policy-makers concerned about the country’s steadily declining population. Compared with other high-income countries, giving birth in Japan is costly to women, both directly in monetary terms and indirectly in terms of the risks of lost income, job loss and career setbacks.^1^ Childbirth is also challenging for women in Japan because the proportion of deliveries with labour analgesia is low compared with other high-income countries.^2^^–^^4^ Perinatal outcomes in Japan remain world-leading. The nation’s 2017 infant mortality rate of 1.9 per 1000 live births and neonatal mortality rate of 0.9 per 1000 live births designates it as one of the safest places in the world to give birth.^5^ Unfortunately these figures mask some of the difficulties faced by Japanese women in accessing the obstetric care and labour analgesia they need.

## Shortage of obstetricians

Japan is lagging behind other countries in gender equality and the role of women in society, politics, education, business and health matters. According to the Global Gender Gap index, the situation is getting worse, with Japan falling from 80th in the world ranking in 2006 to 110th in 2018.^6^ Regarding women’s health, discrimination against female applicants in the entrance examinations of at least nine Japanese medical schools has recently attracted international attention.^7^ Japanese medical facilities have long been understaffed with respect to female physicians in all specialities. According to data from the Organisation for Economic Co-operation and Development (OECD), Japan traditionally has the lowest proportion of female physicians among the most advanced industrialized nations.^8^ According to Japan’s Ministry of Health, Labour and Welfare, only 3667 (36.6%) of the country’s 11 242 obstetricians and gynaecologists are female.^9^ The Japan Society of Obstetrics and Gynecology has boosted recruitment efforts for female obstetricians, and tried to enable existing female obstetricians to keep practising. Nevertheless, half of female obstetricians and gynaecologists currently stop practising to have their own children.^10^ Furthermore, there are few incentives in terms of good career prospects, attractive wages or equitable working environments to encourage students to specialize in women’s medicine.

## Access to birth facilities

Several years ago, Japan’s decreasing number of childbirth facilities became a national talking point, as expectant mothers found it increasingly difficult to find somewhere suitable to deliver their baby safely.^11^ Obstetric services in Japan are provided by hospitals or clinics staffed by obstetric doctors and midwives. Medical facilities with 20 or more inpatient beds are defined as hospitals, and those with fewer than 20 inpatient beds as clinics. Among obstetric hospitals, core facilities are selected and designated as perinatal medical centres by the central government. Most pregnant women give birth at hospitals or clinics and approximately 50 percent of women give birth at private clinics.^12^ The private clinics are usually managed by only a few obstetricians, commonly fifty to sixty years of age.^13^ Due to burdensome working conditions and an ageing workforce, clinics have steadily been closing and most pregnant women now have to go to a hospital for their delivery. Thus, the burden on small- or middle-size birth hospitals has increased, sometimes leading to their closure. In 1996, there were 1818 clinics and 1003 non-perinatal medical centre hospitals, but by 2017 these numbers had fallen to 1350 clinics and 649 non-perinatal medical centre hospitals.^11^ Increasingly, pregnant women in Japan have to travel to large hospitals and in some instances, they may have to travel over 100 km to receive antenatal care and appropriate medical care for delivery.

To prevent the decrease in birth facilities, one possible solution would be to encourage shifting of obstetric tasks to other health workers. However, the work of midwives has historically been severely restricted in Japan; they are not allowed to perform even an episiotomy or a perinatal suture or to prescribe any type of medication. Under these constraints, midwives alone can manage only uncomplicated, low-risk pregnant women. Deliveries at home or at a midwifery centre have become extremely rare; 99.3% (939 518 out of 946 065) of babies are now born in hospitals or clinics.^14^ Some hospitals have tried to maintain a midwifery department where midwives can comprehensively manage deliveries. However, the births that midwives can manage are increasingly limited as maternal age is rising. The mean age of mothers having their first child has increased from 27.6 years of age in 1996 (among 574 043 women) to 30.1 years of age in 2016 (among 459 751 women).^14^^,^^15^ The number of pregnant women with complications, such as preeclampsia and gestational diabetes mellitus, has also been steadily increasing.^16^ Japan urgently needs to train more obstetricians and to ensure they are proficient in providing comprehensive, optimal care for both mother and baby.

## Access to labour analgesia

The prospect of giving birth is an anxious time for most first-time mothers, yet in Japan women know that painkillers are rarely given, if at all. Physicians and other health workers report that more and more women are requesting epidural anaesthesia, but few obstetric centres, hospitals included, are willing or able to provide this care. Furthermore, the cost of 420 000 Japanese yen (3803 United States dollars at the average exchange rate in 2018) that the national health insurance scheme contributes towards having a baby will not cover such an expense.

Up until the mid-18th century, labour pain was considered to be a natural part of the birth process.^17^ The introduction of ether and later chloroform in the 19th century led to labour analgesia becoming increasingly popular among those who could afford it, driven by economic progress in industrializing countries. However, inhalation of systemic analgesic agents during labour was linked to deaths of mothers and neonates,^18^ and the prevalence of labour analgesia therefore remained low until the beginning of the 20th century. After the introduction of epidural analgesia, first used in labour in the 1940s, this safe and effective technique soon became widely adopted where it was available and affordable and infrequently used in developing and resource-poor countries with inadequate health systems.^18^

Labour analgesia can be categorized as either non-pharmacological or pharmacological. Modern, safe and effective techniques, such as epidural infusion of narcotics, local anaesthesia mixtures and patient-controlled analgesia, during and after childbirth are now available. Neuraxial analgesia is now one of the most commonly used analgesic techniques during labour in high-income countries. In 2018 the World Health Organization (WHO) confirmed that “epidural analgesia is recommended for healthy pregnant women requesting pain relief during labour, depending on the woman’s preferences.”^19^ However, only 6.1% of Japanese women (36 849 out of 608 450) are given access to pain relief during delivery, according to a representative study by the health ministry.^20^ This compares with figures of 73.1% (1 920 368 of 2 625 950) in the United States of America^21^^,^^22^ and 83.8% (10 475 of 12 500) in France’s national perinatal survey.^23^ In France, unlike Japan, government incentives to promote childbirth have drastically reversed declines in fertility. In Japan, the annual number of births is continuing to decrease, down to 921 000 in 2018 from 946 065 in 2017.^24^ Although labour analgesia is less common in Asia than in Europe or the United States, the proportion of deliveries with labour analgesia in Japan is low even compared with other countries in Asia: 10% in China, 40% in the Republic of Korea and 60% in Singapore.^2^^–^^4^

For a variety of reasons, some pregnant women in Japan prefer not to use pharmacological interventions for managing labour pain. Instead, they use a wide variety of techniques that attempt to enhance the psycho-emotional and spiritual components of care, such as breathing exercises, massage, acupressure, yoga and hydrotherapy. Using these non-pharmacological approaches, women cope with their labour pain and maintain a sense of personal control over the birth process.^25^ Although in other countries pregnant women can seek physical, emotional and information support before, during and after labour from non-medical supporters known as *doula,*^26^ in Japan pregnant women receive such support from midwives. In this respect, it can be said that labour support in Japan has evolved towards delivery with non-pharmacological care. However, Japan’s unique health-care system, whereby traditional medicine is fully integrated with modern medicine in daily practice and covered by health insurance, may also have an impact. Japanese physicians, all trained in conventional scientific-based medicine, employ both biomedicine and traditional Japanese *kampo* medicine in clinics and university hospitals.^27^^,^^28^ *Kampo* medicine using various herbs is used for a wide variety of conditions, with the prescription rate being highest for disorders associated with pregnancy, childbirth and the puerperium. *Kampo* extracts are known to have fewer and relatively milder effects than biomedical drugs in pregnant and postpartum women. Unfortunately, as a result, pregnant women in Japan who wish to use labour analgesia often cannot get access to it. According to the Japanese Association of Obstetricians and Gynaecologists, only an approximate of 720 out of 2391 hospitals and clinics in Japan offer epidural anaesthesia or combined spinal epidural anaesthesia,^20^ while the health ministry lists only 360 facilities providing labour analgesia in 2019.^29^

## Shortage of anaesthesiologists

The reason why Japanese pregnant women have not used labour analgesia is often explained as being due to cultural beliefs, including the perceived social norm favouring a natural birth. In general, there has been a reluctance to use any medical intervention during normal childbirth because, in the past, both women and obstetricians considered birth as being simply physiological rather than potentially pathological.^30^ This view meant avoidance of any form of anaesthesia or analgesia. Furthermore, many Japanese women, especially the older generations, believe that the mother–child bond is forged and evolves through labour and the pain suffered. Women also fear that pain medication will cause the newborn to be weak and unhealthy, even though women who do not use analgesia for the first child, frequently decide to use it for subsequent births.^31^ More women in Japan are opting to receive neuraxial analgesia during labour, even though the proportion remains low, increasing from 2.6% in 2009 to 6.1% in 2017 according to Japan’s health ministry.^12^^,^^20^ A possible factor in the low uptake of labour analgesia is not so much cultural beliefs as a shortage of anaesthesiologists. According to the World Federation of Societies of Anaesthesiologists, the number of physician anaesthesiologists per 100 000 population in Japan (9.6) is half to one-third the figure for most other OECD countries, such as the United States (20.8), United Kingdom of Great Britain and Northern Ireland (17.9) and France (15.1).^32^ Reflecting Japan’s patriarchal society, only 2260 (31.8%) of the 7107 qualified anaesthesiologists are female.^9^ This situation has been worsened by the lack of task-shifting by existing anaesthesiologists, resulting in a scarcity of health workers who can perform and manage labour analgesia safely.^29^

A temporary solution to Japan’s shortage of anaesthesiologists would therefore be to accelerate plans to delegate anaesthesiologists’ tasks to other trained health workers.^33^ However, this approach is rarely implemented under the current rigid Japanese medical system. For example, Japan has no nurses qualified or authorized to administer anaesthesia. Nurses are not allowed to administer labour analgesia, even after an anaesthesiologist has inserted an epidural catheter. In addition, task-shifting from anaesthesiologists to obstetricians is difficult. As discussed above, midwives or nurses cannot take over obstetricians’ jobs, such as an episiotomy or a perineal suture, and are not allowed to manage delivery comprehensively, even when a pregnant woman is perceived to be at low risk of complications. Consequently, although there seems not to be a shortage of obstetricians and gynaecologists in Japan,^34^ physicians frequently cannot offer to perform labour analgesia. To help ensure a full and comprehensive service is provided to women giving birth in Japan, strict policies are needed to ensure that sufficient numbers of doctors specialize in obstetrics and gynaecology, as well as anaesthesiology. Coupled with a mechanism to ensure a balanced geographical spread of qualified staff, these policies would offer some hope to pregnant women. Unfortunately, this will take time, with 11 years of training needed for qualified obstetricians in Japan.

## Nationwide debate

The scarcity of anaesthesiologists represents a threat to safe and pain-free childbirth in Japan, especially as about half of all births occur in private clinics.^12^ These commercial clinics do offer labour analgesia. However, clinics are often understaffed, with only a few obstetricians, who often resort to performing neuraxial anaesthesia by themselves. Moreover, due to the scarcity of anaesthesiologists, most deliveries with neuraxial anaesthesia are elective procedures, which may require abnormal use of labour induction and instrument-assisted delivery, increasing the risks of abnormal uterine bleeding.^12^

The gravity of the current situation is demonstrated by the fact that since 2010, there have been 14 cases in Japan in which the use of anaesthesia and pain relief in labour and delivery resulted in the death or serious complications for the mother and/or infant.^12^ These incidences have attracted widespread attention, stimulating intense debate. Among these 14 deaths, only one was directly related to labour analgesia. All other deaths were due to bleeding, embolism and infection. Although eight deaths happened in private clinics, considering that about a half of deliveries with labour analgesia are managed in such clinics,^12^ we could not confirm that deliveries with labour analgesia in private clinics were at particularly high risk. However, the Japanese Association of Obstetricians and Gynecologists reported that emergency care or transport of patients to a higher-level facility might have been delayed in several deaths since those private clinics were short-staffed and usually did not store blood for transfusions.^12^ The association has since clarified the risks of labour analgesia, stressing that neuraxial anaesthesia during delivery did not increase the maternal mortality rate in Japan. However, evidence from the United States indicates that maternal death and severe morbidity can arise in any setting where physicians and other health workers caring for women in labour and providing analgesia have not received life-support training, planning and experience.^35^ A lack of qualified staff and facilities has also been recognized as a factor behind the proportion of caesarean section deliveries in Japan rising to 23.2% (10 761 out of 46 451 deliveries), well above the WHO-recommended rate of no more than 10–15%.^9^

To improve the quality of obstetric anaesthesia, the Japan Society for Obstetrics and Perinatology plans to issue qualifications for medical staff providing labour analgesia. However, the society will limit the requisite qualifications to obstetricians and anaesthesiologists and thus the problem of a shortage of human resources will remain. In Japan no suitable and sustainable solution has yet been proposed to improve both the safety and availability of labour analgesia, meaning that women will continue to suffer during childbirth.

## Conclusion

Globally, women’s health issues are increasingly becoming a higher priority. According to the Japanese government’s latest 4th Basic Plan for Gender Equality,^36^ the current policy aims to make gender equality the most important issue in Japan by 2020 and to make the country internationally recognized for its gender impartiality and equal opportunity. All current evidence points to these goals being unrealistic. Nevertheless, Japan must accelerate efforts to improve gender equality in all areas and especially in health matters. This includes ensuring full and unrestricted access to prenatal care, resources and expertise, as well as access to safe, pain-free childbirth and comprehensive medical care before, during and after birth.

In 2015, the Japan Society of Obstetrics and Gynecology announced major plans to renovate and modernize obstetrics and gynaecology services nationwide.^37^ However, considering that the number of obstetrics facilities, especially small- or middle-size facilities in rural areas, have steadily decreased, simply intensifying work at these facilities will not solve the problem and could impair access to perinatal care. Provision of optimal care for both mothers and babies will necessitate access to appropriate medical institutions and expert care. Consequently, Japan needs to overhaul and remodel its obstetrics facilities, reorganize the regional perinatal care system,^11^ promote task-shifting, train more obstetricians and anaesthesiologists and better integrate medical and non-medical care for pregnant women. If this is not done, the nation will be failing in its duties and responsibilities, accelerating Japan’s move down the Global Gender Gap index. At the same time Japan risks failing to meet its commitments to the sustainable development goals, to its own policy targets and worse still, increasing the neglect of the rights, health and equality of women in the country.
